# Why d-Mannose May Be as Efficient as Antibiotics in the Treatment of Acute Uncomplicated Lower Urinary Tract Infections—Preliminary Considerations and Conclusions from a Non-Interventional Study

**DOI:** 10.3390/antibiotics11030314

**Published:** 2022-02-25

**Authors:** Florian Wagenlehner, Horst Lorenz, Oda Ewald, Peter Gerke

**Affiliations:** 1 Clinic and Polyclinic for Urology, Pediatric Urology and Andrology, Justus-Liebig-University Giessen, 35392 Giessen, Germany; florian.wagenlehner@chiru.med.uni-giessen.de; 2 BBS-Büro für Biometrie und Statistik, Im Unterfeld 17, 63543 Neuberg, Germany; horst.lorenz@bbs-neuberg.de; 3 MCM Klosterfrau Vertriebsgesellschaft mbH, Gereonsmuehlengasse 1-11, 50670 Cologne, Germany; oda.ewald@klosterfrau.de

**Keywords:** urinary tract infection, cystitis, d-mannose, antibiotics, acute cystitis symptom score

## Abstract

Urinary tract infections (UTIs) are very frequent in women and can be caused by a range of pathogens. High recurrence rates and increasing antibiotic resistance of uropathogens make UTIs a severe public health problem. d-mannose is a monosaccharide that can inhibit bacterial adhesion to the urothelium after oral intake. Several clinical studies have shown the efficacy of d-mannose in the prevention of recurrent UTIs; these also provided limited evidence for the efficacy of d-mannose in acute therapy. A recent prospective, non-interventional study in female patients with acute cystitis reported good success rates for treatment with d-mannose. Here, we present data from a post hoc analysis of this study to compare the cure rate of d-mannose monotherapy with that of antibiotics. The results show that d-mannose is a promising alternative to antibiotics in the treatment of acute uncomplicated UTIs in women.

## 1. Epidemiology and Infectiology of Acute Uncomplicated Urinary Tract Infections

Bacterial urinary tract infections (UTIs) are among the most common infectious diseases, affecting 150 million people each year worldwide [1]. Although the disease affects both sexes, it is much more prevalent in women [2,3]. Based on certain characteristics, UTIs can be divided into different subcategories. Lower UTIs (cystitis) and upper UTIs (pyelonephritis) can be differentiated according to the anatomical region affected [2,4]. Lower UTIs can be subdivided clinically into uncomplicated and complicated types. Since UTIs in men are considered to be complicated, uncomplicated lower acute UTIs (acute uncomplicated cystitis, AUC) typically occur in healthy women without structural or neurological urinary tract abnormalities [2,4]. Risk factors for developing cystitis are female gender, prior UTI, sexual activity, vaginal infection, UTI history during premenopause or in childhood, family history, diabetes, obesity and genetic susceptibility, among others [2,4,5,6].

UTIs can be triggered by various microorganisms, including Gram-negative and Gram-positive bacteria as well as certain fungi. *Escherichia coli* (*E. coli*) are the most common cause of uncomplicated as well as complicated UTI [7]. Further pathogens involved in acute infections include *Klebsiella pneumoniae*, *Staphylococcus saprophyticus*, *Enterococcus faecalis*, group B *Streptococcus* (GBS), *Proteus mirabilis*, *Pseudomonas aeruginosa*, *Staphylococcus aureus* and *Candida* spp. [7].

## 2. Antibiotics as Current Standard Therapy and Relevance of Resistance Development

Since UTIs have mainly bacterial etiologies, the crucial role of antibacterial treatments is unquestionable. According to international guidelines, antibiotic therapy with fosfomycin trometamol, pivmecillinam or nitrofurantoin is recommended as first-line therapy for uncomplicated UTI [4,8]. Although uncomplicated UTIs are often self-resolving (cure rates of 15–45%), almost all UTIs are treated with antibiotics [4]. UTIs are usually treated empirically, without analysis of the exact bacterial etiology or antibiotic susceptibilities [9]. Besides their positive properties, antibiotics also have some drawbacks. In addition to long-term alteration of the normal microbiota in the vagina and gastrointestinal tract, the emergence of multidrug-resistant microorganisms has played an increasingly important role in recent years [10,11,12]. Moreover, despite appropriate antibiotic therapy, 20–30% of women with an initial UTI will develop a recurrent infection within 4–6 months [13]. Recurrent UTI (rUTI) is commonly defined by at least three episodes per year or at least two episodes within six months [2]. Repeated administration of antibiotics to treat rUTIs often results in the development of resistance to the antibiotics that were originally effective [11,14]. The high recurrence rates of UTIs and increasing antimicrobial resistance among uropathogens are a severe public health threat with a significant burden on healthcare system resources and patients’ quality of life [12,15,16]. Thus, the growing resistance to antibiotic therapies highlights the urgent need to develop alternative treatment strategies to fight UTIs [11,17].

## 3. Fundamental Aspects of the d-Mannose Mode of Action

**Figure 1:**
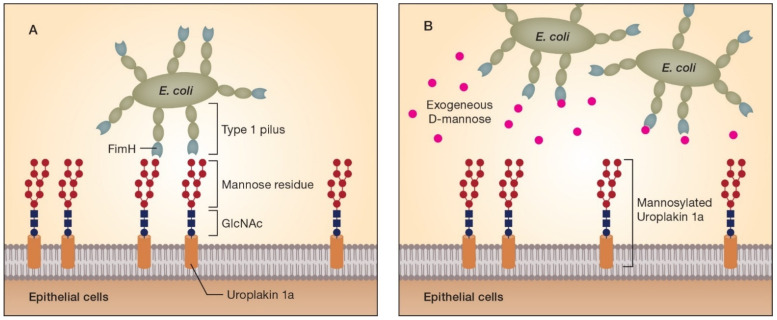
Exogenous d-mannose in UTI: mode of action scheme. (**A**) Adherence of uropathogens depends on binding of the adhesin, e.g., FimH protein (located at the tips of the bacteria’s type 1 pili) to mannosylated proteins, such as uroplakin 1a, located on the epithelial cell surface. (**B**) Exogenously delivered d-mannose can prevent adhesion of *E. coli* by saturating the FimH binding sites. Thus, d-mannose competitively inhibits adhesion of bacteria to the urothelium and facilitates their clearance by urine flow. GlcNAc: *N*-acetylglucosamine.

Adhesion of pathogens to the urothelial cells prevents their removal or washing off, and is therefore their first step in colonizing the urinary bladder. Accordingly, this represents a crucial step in the onset of infection, and strategies interfering with bacterial adhesion can prevent or treat UTIs [3,18]. Uropathogens entering the bladder possess various virulence factors including fimbriae or pili, which play an integral role in attachment and colonization of the urinary tract [19]. Type 1 pili of *E. coli*—the most common UTI-causing pathogen—are composed of Fim proteins with adhesin FimH located on the tips of these structures. Adhesins are capable of interacting with various host structures, such as peptides or glycosylated residues on the epithelial cell surface and in the extracellular matrix [3]. Besides adhesion to the host cell, adhesins are also involved in biofilm formation, antimicrobial resistance and internalization of the pathogen into the host cells [3]. FimH enables the bacteria to bind onto bladder epithelial cells by attaching to uroplakin 1a, which consists of glycosylated proteins with mannose molecules as terminal units (Figure 1A) [20,21]. Since it has been shown in UTI mouse models that FimH is critical for the pathogenesis of *E. coli* in AUC and is conserved in various *E. coli* strains [22], this adhesin is considered to be a good target for therapeutic interventions [23].

D-mannose is an epimer of d-glucose with structural similarities to the mannose residues on the urothelium surface or transmembrane proteins such as uroplakin 1a. Thus, adhesion of *E. coli* via type 1 pili can be inhibited by exogenous d-mannose saturating FimH adhesins (Figure 1B) [17,18]. This facilitates clearance of the pathogens by the urine flow.

## 4. Clinical Data for d-Mannose in UTI

Several clinical studies have investigated the effects of d-mannose monotherapy in the treatment of AUC and prevention of recurrent UTIs. In a prospective, non-comparative study, Domenici et al. analyzed the efficacy of d-mannose in the treatment of acute uncomplicated UTI and its utility in the management of recurrences in 43 women [18]. The authors observed a significant improvement of most UTI symptoms following administration of 1.5 g d-mannose, twice daily for three days and then once a day for 10 days. Patients were then randomized into two groups, one receiving prophylaxis with d-mannose administered once a day for a week every other month for six months, and the other without d-mannose administration. The recurrence rate for women receiving prophylaxis (4.5%) was significantly lower than in the untreated group (33.3%; *p* = 0.05). The mean time to onset of UTI was 43 days (±4.1 standard deviations (SD)) in the prophylaxis group, and 28 days (±5.4 SD) in the other group (*p* = 0.0001). No side effects were reported, even during long-term administration [18].

To evaluate the efficacy of d-mannose in the treatment and prophylaxis of rUTIs, Porru et al. performed a randomized cross-over trial including 60 women with acute symptomatic UTI and three or more rUTIs during the preceding 12 months [24]. Patients were randomly assigned to antibiotic treatment with trimethoprim/sulfamethoxazole (TMP-SMZ: 2 × 160 mg/800 mg daily for 5 days) or therapy with d-mannose (3 × 1 g daily for 2 weeks), followed by prophylactic therapy over 5 months with TMP-SMZ (1 × 160 mg/800 mg daily for one week each month) or d-mannose (1 × 1 g daily). In the group treated with d-mannose, a significantly increased mean time to UTI recurrence (200 days) was observed in comparison to the antibiotic group (52.7 days; *p* < 0.0001). Porru et al. stated that no significant side effects limiting a long-term treatment with d-mannose have been reported [24].

In a randomized controlled trial, Kranjčec et al. investigated the efficacy of d-mannose in rUTI prevention [25]. After initial antibiotic treatment of AUC, 308 women were randomly divided into three groups receiving daily treatment with 2 g d-mannose, 50 mg nitrofurantoin or no prophylaxis. Recurrence rates were 14.6% in the d-mannose group, 20.4% in the nitrofurantoin group and 60.8% in the group without prophylaxis. The number of rUTIs was significantly higher in the non-prophylaxis group than in both treated groups (*p* < 0.001). d-mannose significantly reduced the risk of rUTI, which was comparable to that in the nitrofurantoin group. In 7.8% of patients receiving d-mannose prophylaxis, diarrhea was reported as an adverse event. However, patients in the d-mannose group had a significantly lower risk of side effects (relative risk 0.276, 95% confidence interval (CI) 0.132–0.574, *p* < 0.0001) than in the nitrofurantoin group (27.2%, usually diarrhea, nausea and vaginal burning) [25].

An open-label prospective feasibility study by Phe et al. evaluated d-mannose for the prevention of UTIs in 22 patients with multiple sclerosis [26]. The authors showed an association between administration of d-mannose and a significant decrease in the number of monthly proven UTIs. No adverse events were reported in patients with multiple sclerosis who were treated with d-mannose [26].

In 2020, Lenger et al. published a systematic review and meta-analysis of the potential of d-mannose to reduce UTI recurrence in adult women with a history of rUTI and compared its efficacy with that of a placebo or other preventive agents [27]. Comparison of d-mannose with placebo resulted in a pooled relative risk for rUTI of 0.23 (95% CI 0.14–0.37; heterogeneity = 0%; d-mannose: n = 125, placebo: n = 123). In addition, the pooled relative risk of rUTI comparing d-mannose with preventive antibiotics was 0.39 (95% CI 0.12–1.25; heterogeneity = 88%; d-mannose: n = 163, antibiotics: n = 163) [27]. These data show that d-mannose can provide protection against rUTI with an efficacy similar to that of antibiotics.

## 5. Clinical Diagnosis and Assessment of Treatment Efficacy in UTIs with a Validated Measuring Instrument

AUC is typically diagnosed based on clinical symptoms, such as a painful or burning sensation when urinating (dysuria) or frequent urination (pollakiuria) in the absence of abnormal vaginal irritation/discharge [2,4,28]. However, the definitions for clinical diagnosis of AUC and assessment of clinical healing after treatment vary widely between clinical studies. The acute cystitis symptom score (ACSS) is derived from a diagnostic questionnaire that measures patient-reported outcomes in women with AUC, and assesses the symptoms and their impact on the quality of life [29]. It has been clinically validated in several languages, including German and English (http://www.acss.world/downloads.html (accessed on 3 February 2022)). The ACSS has been proven to be suitable for the clinical diagnosis of AUC in women and in monitoring treatment success during and after therapy [30,31,32,33,34]. Furthermore, it has already been used as a “patient-reported outcomes measure” (PROM) in several clinical studies [31,32,35,36,37,38].

The ACSS subcategory “typical symptoms” (referred to below as “typical” domain) contains six patient-reported items: urination frequency, urination urgency, dysuria, suprapubic pain, incomplete bladder emptying and visible blood in the urine. Symptom intensity is rated with a four-point Likert scale (0 = none, 1 = mild, 2 = moderate, 3 = pronounced symptoms) [29]. A summary score of ≥6 in the “typical” domain indicates a clinically diagnosed AUC [29,39].

For a clinical diagnosis of AUC, the current guidelines of the US Food and Drug Administration (FDA) and the European Medicines Agency (EMA) recommend the presence of at least two of four signs or symptoms (i.e., dysuria, urinary frequency, urinary urgency and suprapubic pain) [40], or a minimum number of symptoms such as urinary frequency, urinary urgency and dysuria, respectively [41]. Thus, the ACSS “typical” domain is compatible with the recommendations of public authorities for AUC diagnosis [35]. Therefore, the ACSS can be recommended for epidemiological and interventional studies.

## 6. Post Hoc Analysis of the Potential Efficacy of d-Mannose in the Treatment of Acute Episodes of UTI

### 6.1. Clinical Efficacy of d-Mannose in the Treatment of Acute UTI—A Non-Interventional Study

Recently, Wagenlehner et al. assessed the suitability of a d-mannose-containing product as therapy for women with an acute episode of uncomplicated UTI in a multicenter, prospective, non-interventional study (NIS) [42]. The original study evaluated 97 patients who were divided into three groups after completion of the study according to the applied treatment: d-mannose monotherapy, d-mannose in combination with antibiotics, and the combination of d-mannose with other therapeutic measures (most frequently kidney and bladder tea). During the first three days, d-mannose (2 g) was applied three times a day followed by twice daily at days 4 and 5. In the course of the study, patients rated five symptoms as present/absent or on a four-/six-point Likert scale until they were subjectively free of symptoms, but for a maximum of seven days: (1) burning sensation/pain while urinating, (2) frequency of bladder emptying, (3) small amounts of urine, (4) constant urge to urinate and (5) change in urine. Healing was defined as relief from burning/pain during urination (score reduction to 0 or 1 on day 7 or on the last day of documentation). A patient was considered to be free of symptoms if burning/pain had a score of “0” or “1”, urination frequency was rated as “not more frequent” or “hardly more frequent” and all other symptoms were rated with “0” or “not existing” [42].

After three days, 85.7% of the patients under d-mannose monotherapy were assessed as healed compared to 56.6% in the group treated with d-mannose and antibiotics and 56.3% in the group combining d-mannose with other measures. On the last day of documentation, healing rates were largely comparable between the subgroups (92.9% vs. 83.0% or 87.5%). Furthermore, the proportion of patients considered to be free of symptoms was higher in the monotherapy group (78.6%) than in the groups combining d-mannose with antibiotics (67.9%) or other measures (62.5%) [42].

Seven (7.2%) of 97 patients evaluated in the NIS reported a total of 10 adverse events of mild or moderate severity. The most common side effects were gastrointestinal complaints and in one case a skin rash was reported. According to the physician’s assessment, in seven events a causal relationship with the use of d-mannose was possible or could not be excluded. Six of these seven events occurred in combination with an antibiotic, and one (flatulence) together with other therapeutic measures [42].

### 6.2. Transfer of Clinical Data of the NIS to the ACSS as a Validated Instrument

For post hoc data analysis, the clinical symptoms assessed in the NIS were transferred to the “typical” domain of the ACSS. Items, which were assessed as present or absent in the NIS, were rated with “2” or “0” for the transfer, respectively. The rating of the item assessed by a six-point scale in the NIS was transferred to a four-point scale as used in the ACSS (Table 1). Except for the ACSS item “suprapubic pain”, all symptoms of the ACSS were also investigated in the NIS. In order to balance the overall score range of the NIS (5 items) with that of the ACSS (6 items), the leading AUC symptom “burning/pain during urination” was rated twice for transfer to the ACSS. This approach was required to generate a balanced total score from the NIS data, which then also consisted of 6 item values as in the ACSS.

For inclusion in the post hoc analysis, NIS patients needed to meet the following criteria: (i) AUC diagnosed by a physician, (ii) follow-up examinations after diagnosis and (iii) a summary score of the “typical” domain ≥ 6 at the beginning of the study (corresponding to the ACSS-based definition of AUC) [36]; this procedure reduced the number of patients who could be evaluated. Based on the above clinical symptoms, the summary score of the “typical” domain was calculated for each patient at each study day.

Alidjanov et al. defined and evaluated six different thresholds using the ACSS “typical” domain or a combination of the domains “typical” and “quality of life” to specify “clinical cure” during treatment of AUC. In addition, two further thresholds were based on the criteria of the FDA and EMA guidelines. From these eight definitions the following threshold “B” was chosen for our analysis, since its criteria best matched the data available from the NIS: a summary score of “typical” domain ≤4 and “no visible blood in urine” [36]. In addition, the threshold was applied in a modified form (score ≤ 2 and “no visible blood in urine”) to gain a second, more robust estimate for the clinical cure rate. For both estimates, the corresponding 95% CI was determined. The last documented score value of each patient was pursued by “last observation carried forward”.

### 6.3. Estimated Cure Rates from Reanalysis of the NIS with d-Mannose Treatment for Acute UTI

Finally, to determine the efficacy of d-mannose in the treatment of AUC, we analyzed two patient groups according to the treatment documented in the patient diaries. One group included patients treated with d-mannose as monotherapy. To increase statistical precision for the efficacy estimates, this cohort was expanded in the second group to further include patients who were taking other measures in addition to d-mannose except antibiotics. After applying the above inclusion criteria (see Section 6.2), 23 patients treated with d-mannose monotherapy were evaluated with post hoc analysis. In the second group, 13 patients under d-mannose and additional non-antibiotic measures were added to these 23, making a total of 36 patients analyzed in this group, referred to below as d-mannose and other measures.

**Figure 2:**
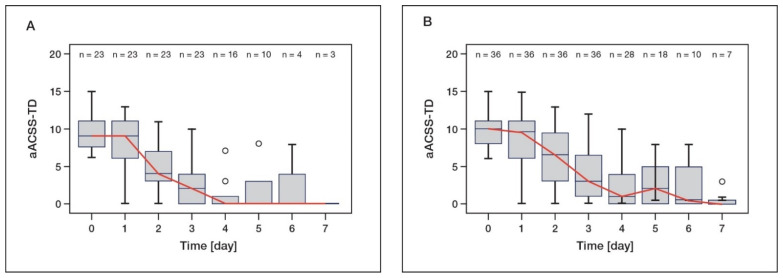
Median aligned ACSS of “typical domain” (aACSS-TD) over 7 days for patients receiving (**A**) d-mannose monotherapy or (**B**) d-mannose and other measures except antibiotics. Clinical symptoms assessed in the NIS were transferred to the “typical domain” of the ACSS. The pooled summary score of the treated cohort is shown for each day as a box plot. The red line indicates the median aACSS-TD.

In patients treated with d-mannose only, the median of the aligned ACSS “typical domain” (aACSS-TD) had already decreased from 9.0 at baseline to 2.0 on day 3 (Figure 2A). The median aACSS-TD was 0.0 from day 4. Patients treated with d-mannose and other measures showed a clear decrease in the median aACSS-TD from 10.0 before the start of treatment to 3.0 on day 3 (Figure 2B). The group reached a median score of 0.0 at day 7. Overall, the course of aACSS-TD reduction was similar in both groups.

According to the aACSS-TD using threshold “B” (as defined by Alidjanov et al. 2020: score ≤ 4 and “no visible blood in urine”), d-mannose monotherapy achieved an estimated cure rate of 91.3% (95% CI 72–99%; Table 2). The calculated cure rate for d-mannose and other measures was 86.1% (95% CI 71–95%; Table 2).

For the more robust estimate (modified threshold “B”: score ≤ 2 and “no visible blood in urine”), the cure rate for patients receiving d-mannose monotherapy was 87.0% (95% CI 66–97%; Table 2). For patients treated with d-mannose and other measures, a cure rate of 77.8% (95% CI 61–90%) was computed (Table 2).

Furthermore, to differentiate the treatment effect for patients with d-mannose monotherapy (n = 23) and patients with d-mannose plus other non-antibiotic treatments (n = 13), Kaplan–Meier estimates were determined for the course of the aACSS-TD. The probability (*p*) of reaching an aACSS-TD of zero after day 4 was 0.48 (95% CI 0.21; 0.66) and 0.25 (95% CI 0.0; 0.46) in patients under d-mannose monotherapy compared to d-mannose plus other non-antibiotic treatment, respectively. On day 5, the probabilities were 0.74 (95% CI 0.45; 0.88) and 0.44 (95% CI 0.06; 0.66), respectively. This means there were comparable results for both treatment groups, with a trend to better results (higher *p*-values) in the monotherapy group.

## 7. Cure Rates of Controlled Trials—Antibiotic Treatment of Acute UTI

Success rates, odds ratios and/or other risk measures have been published to estimate the effect of antibiotic treatment, mainly in terms of microbiological response. To illustrate and compare these results, simple success rates (healing rates) of the published data were calculated and supplemented with the corresponding 95% CIs as presented below [43].

For example, a comprehensive meta-analysis including 27 trials in about 1700 patients with cystitis compared the efficacy of fosfomycin with other antibiotics [44]. This meta-analysis found no difference between fosfomycin and comparators regarding microbiological success. The cure rate for fosfomycin was 83.8% (95% CI 81.2–86.3%) and for nitrofurantoin it was 80.9% (95% CI 73.9–86.7%). For other antibiotics, the overall cure rate was 83.7% (95% CI 80.9–86.3%) [44].

A more recent meta-analysis of 12 studies obtained estimates of the microbiological response rates and corresponding 95% CIs for nitrofurantoin and placebo treatment [45]. For patients with AUC under placebo, an overall microbiological response of 34.2% (95% CI 28.8–39.7%) was calculated. Patients treated with nitrofurantoin (n = 934) achieved an overall microbiological response of 76.6% (95% CI 66.5–86.7%) [45].

As the clinical cure or improvement of AUC symptoms during and following treatment becomes increasingly focused on treatment efficacy, the clinical success rate should be evaluated primarily in clinical trials. A systematic review of 19 studies including 3779 patients in total compared the clinical and microbiological efficacy of single-dose fosfomycin with other antibiotic regimens [46]. The microbiological cure rate was 78.9% (95% CI 76.2–81.4%) for fosfomycin and 77.1% (95% CI 72.2–81.5%) for nitrofurantoin. The mean effect of other antibiotics evaluated in this publication resulted in a cure rate of 81.6% (95% CI 78.0–84.9%). The clinical success rates were 77.9% (95% CI 75.4–80.3%) for fosfomycin and 79.0% (95% CI 75.0–82.6%) for nitrofurantoin. The averaged clinical cure rate for the other antibiotics was 84.6% (95% CI 81.2–87.6%) [46]. These results showed no clinically significant differences between the microbiological and clinical cure rates and gave comparable results between the different antibiotic groups.

**Figure 3:**
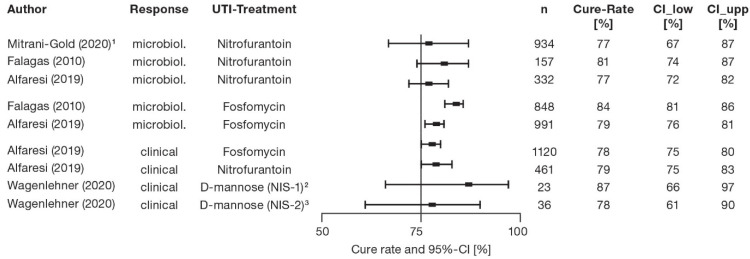
Cure rates of patients with AUC treated with nitrofurantoin, fosfomycin or d-mannose [42,44,45,46]. Vertical line indicates cure rate of 75%. Squares indicate range from 1st to 3rd quartile of estimated cure rates. Horizontal lines indicate 95% CIs. ^1^ Random effect model. ^2^ d-mannose monotherapy (for numbers, see Table 2). ^3^ d-mannose and other measures except antibiotics (for numbers, see Table 2).

Overall, these analyses reveal comparable clinical cure rates under treatment with fosfomycin, nitrofurantoin and other antibiotics, which are on the same level as those estimated for treatment with d-mannose monotherapy by post hoc analysis of the NIS study (Figure 3).

## 8. Time-Dependent Changes of Symptoms in AUC Patients Treated with d-Mannose or Antibiotics

**Figure 4:**
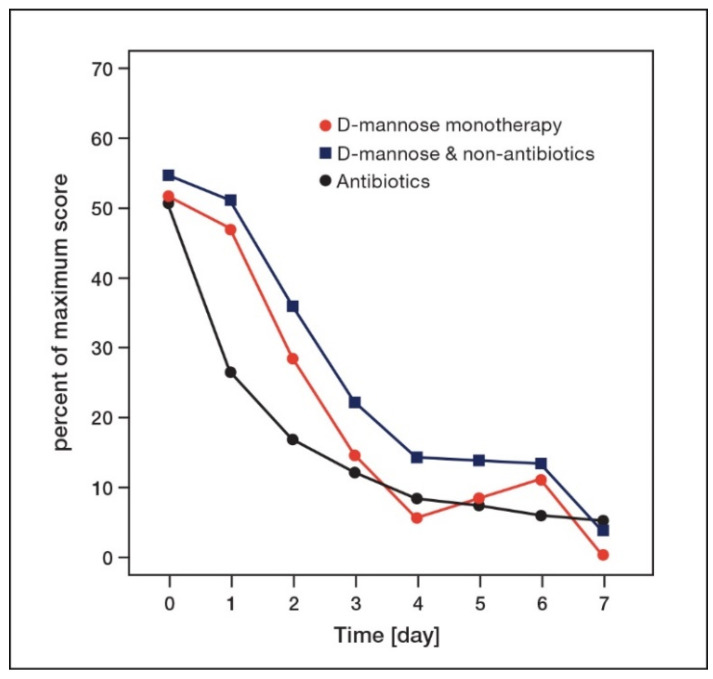
Time course of mean symptom scores in AUC over 1 week in patients receiving d-mannose compared to median symptom score in patients receiving antibiotics (for data, see Table 3).

Besides assessing treatment efficacy as described by cure rate, time to symptom improvement also plays an important role in the patients’ quality of life. In order to compare time to symptom improvement under treatment with d-mannose and antibiotics, we searched for published studies using antibiotics that recorded a clinical summary symptom score in patients with AUC over time. However, the scores were often weighted differently, so we had to perform some adaptation to achieve comparability between the various studies. The maximum value achievable in each symptom score was set to 100%, and the score values reported were then calculated as the corresponding proportion. The resulting relative (or normalized) daily mean scores were compared between d-mannose (based on aACSS-TD from the post hoc analysis) and various antibiotic treatments (Table 3) [38,42,47,48,49,50,51,52]. In addition, we compared the median values from groups receiving antibiotic treatments and mean values of d-mannose monotherapy or d-mannose and other non-antibiotic measures (Figure 4).

The normalized symptom scores of the different treatments in the individual studies were comparable at baseline, with most scores ranging from 46% to 56%, also including the two d-mannose groups (Table 3). At day 3, a distinct decrease in the normalized total symptom scores to 10–25% was observed in all treatment groups considered. After a period of 7–8 days, the normalized total symptom score was lowered to 0–12% in all treatments.

Overall, a clear trend regarding symptom relief over time was found with the various treatments. The normalized symptom score under treatment with d-mannose monotherapy decreased from 51.7% at baseline to 5.6% at day 4, while with d-mannose and other non-antibiotic measures, it decreased from 54.5% to 13.9%. Likewise, with d-mannose, the median value derived from all antibiotic treatments decreased from 51% at baseline to 8.2% at day 4. In addition, the time-dependent symptom reduction after 3 days of treatment was similar following d-mannose monotherapy and antibiotic treatments, which again underlines the potential of d-mannose in the treatment of AUC (Figure 4).

## 9. Conclusions

Our post hoc analysis shows that patients using d-mannose as monotherapy in AUC achieved very good clinical cure rates, similar to those achieved by patients receiving antibiotic treatments. Furthermore, symptom relief after 3 days of treatment was also comparable between d-mannose monotherapy and antibiotics. These findings are in line with previous studies showing similar effectiveness of d-mannose to that of antibiotics in UTI prevention. Therefore, d-mannose may be a safe and effective alternative to antibiotics in the treatment of AUC. However, further randomized, controlled trials including a relevant number of patients are necessary, in order to confirm the beneficial effect of d-mannose in AUC.

## Figures and Tables

**Table 1:** Transfer of clinical symptoms from Wagenlehner et al. (2020) to ACSS.

Symptom	Original Assessment	Rules for Transfer to ACSS “Typical Domain”
Urination frequency	0–3	0–3 (no adaptation necessary)
Urination urgency	No, yes	No ⇒ 0; yes ⇒ 2
Urination burning/pain (rated twice)	0–5	0 ⇒ 0; 1 ⇒ 1; 2, 3 ⇒ 2; 4, 5 ⇒ 3
Incomplete bladder emptying	No, yes	No ⇒ 0; yes ⇒ 2
Visible blood in urine	No, yes	No ⇒ 0; yes ⇒ 2

**Table 2:** Time course of the summary aligned ACSS “typical” domain (aACSS-TD) and the estimated cure rates of patients receiving d-mannose monotherapy or d-mannose and other measures.

	Group 1: d-Mannose Monotherapy (n = 23)			Group 2: d-Mannose and Other Measures (n = 36)		
Day	aACSS-TD (Median)	Cure Rate ^1^	Cure Rate ^2^	aACSS-TD (Median)	Cure Rate ^1^	Cure Rate ^2^
0	9.0	-	-	10.0	-	-
1	9.0	4.4%	4.4%	9.5	5.6%	2.8%
2	4.0	52.2%	4.4%	6.5	38.9%	5.6%
3	2.0	73.9%	56.5%	3.0	61.1%	41.7%
4	0.0	91.3%	82.6%	1.0	80.6%	61.1%
5	0.0	91.3%	82.6%	2.0	80.6%	66.7%
6	0.0	91.3%	87.0%	0.5	86.1%	77.8%
7	0.0	91.3%	87.0%	0.0	86.1%	77.8%
Cure rate [95% CI] on day 7	-	91.3% [72–99%]	87.0% [66–97%]	-	86.1% [71–95%]	77.8% [61–90%]

^1^ Score ≤ 4 and “no visible blood in urine”; ^2^ score ≤ 2 and “no visible blood in urine”.

**Table 3:** Time course of mean symptom score normalized by maximum of the individual scales [%] over 1 week in AUC patients receiving d-mannose or antibiotic treatments.

Treatment [Reference]	Baseline	Day 1	Day 2	Day 3	Day 4	Day 5	Day 6	Day 7–8
d-mannose monotherapy ^1^	51.7	46.9	28.5	14.5	5.6	8.3	11.1	0
d-mannose and other measures ^1^	54.5	50.9	35.9	21.9	13.9	13.6	13.3	3.2
Fosfomycin (single dose) ^1^ [38]	56.1	-	-	25.0	-	-	-	11.7
Fosfomycin (single dose) ^2^ [47]	50.8	26.7	16.7	10.0	8.3	7.5	5.8	4.2
Pivmecillinam (5 days) ^3^ [52]	42.6	26.7	14.0	12.0	8.0	7.3	7.3	6.7
Pivmecillinam (3 days) ^1^ [49]	68.3	41.7	22.2	13.9	5.6	5.0	3.9	-
Ciprofloxacin (3 days) ^4^ [50]	48.3	-	-	-	10.8	-	-	5.0
Ciprofloxacin (5 days) ^3^ [51]	50.7	-	-	-	-	-	-	10.0
Norfloxacin (3 days) ^5^ [48]	46.0	-	-	11.6	-	-	-	4.0%
Summary of antibiotic treatment (median)	50.7	26.7	16.7	12.0	8.2	7.3	5.8	5.0

^1^ 6 items on scale 0–3 (max. = 18), ^2^ 3 items on scale 0–4 (max. = 12), ^3^ 5 items on scale 0–3 (max. = 15), ^4^ 4 items on scale 0–3 (max. = 12), ^5^ 5 items on scale 0–6 (max. = 30). d-mannose groups: aACSS-TD generated from NIS data; antibiotic treatments: data from controlled clinical studies [38,47,48,49,50,52] and one open clinical study [51].

## Data Availability

Further data regarding post hoc analysis of the NIS are available from the sponsor by request.
